# The role of dendritic cells in tertiary lymphoid structures: implications in cancer and autoimmune diseases

**DOI:** 10.3389/fimmu.2024.1439413

**Published:** 2024-10-11

**Authors:** Mariana Reste, Kristi Ajazi, Ayca Sayi-Yazgan, Radmila Jankovic, Biljana Bufan, Sven Brandau, Espen S. Bækkevold, Florent Petitprez, Malin Lindstedt, Gosse J. Adema, Catarina R. Almeida

**Affiliations:** ^1^ Institute of Biomedicine (iBiMED), Department of Medical Sciences, University of Aveiro, Aveiro, Portugal; ^2^ Department of Immunotechnology, Lund University, Lund, Sweden; ^3^ Department of Molecular Biology and Genetics, Faculty of Science and Letters, Istanbul Technical University, Istanbul, Türkiye; ^4^ Department of Life Sciences, Centre for Inflammation Research and Translational Medicine, College of Health and Life Sciences, Brunel University London, Uxbridge, United Kingdom; ^5^ Faculty of Medicine, Institute of Pathology, University of Belgrade, Belgrade, Serbia; ^6^ Department of Microbiology and Immunology, University of Belgrade - Faculty of Pharmacy, Belgrade, Serbia; ^7^ Experimental and Translational Research, Department of Otorhinolaryngology, University Hospital Essen, Essen, Germany; ^8^ Department of Pathology, Oslo University Hospital-Rikshospitalet, Oslo, Norway; ^9^ Centre for Reproductive Health, Institute for Regeneration and Repair, University of Edinburgh, Edinburgh, United Kingdom; ^10^ Radiotherapy & OncoImmunology Laboratory, Department of Radiation Oncology, Radboud University Medical Center, Nijmegen, Netherlands

**Keywords:** tertiary lymphoid structures (TLS), tertiary lymphoid organs (TLO), dendritic cells (DC), anti-tumor immunity, autoimmunity

## Abstract

Tertiary Lymphoid Structures (TLS) are organized aggregates of immune cells such as T cells, B cells, and Dendritic Cells (DCs), as well as fibroblasts, formed postnatally in response to signals from cytokines and chemokines. Central to the function of TLS are DCs, professional antigen-presenting cells (APCs) that coordinate the adaptive immune response, and which can be classified into different subsets, with specific functions, and markers. In this article, we review current data on the contribution of different DC subsets to TLS function in cancer and autoimmunity, two opposite sides of the immune response. Different DC subsets can be found in different tumor types, correlating with cancer prognosis. Moreover, DCs are also present in TLS found in autoimmune and inflammatory conditions, contributing to disease development. Broadly, the presence of DCs in TLS appears to be associated with favorable clinical outcomes in cancer while in autoimmune pathologies these cells are associated with unfavorable prognosis. Therefore, it is important to analyze the complex functions of DCs within TLS in order to enhance our fundamental understanding of immune regulation but also as a possible route to create innovative clinical interventions designed for the specific needs of patients with diverse pathological diseases.

## 1 Introduction

Tertiary Lymphoid Structures (TLS) are organized aggregates of immune cells that arise postnatally in nonlymphoid tissues (1). They comprise both T/B lymphocytes as well as professional antigen-presenting cells, most notably Dendritic Cells (DCs). These structures are formed in response to cytokines and chemokines produced by fibroblasts and are found in inflamed, infected, or malignant tissues (2). Given their proximity to disease loci, and presumed impact on disease development, TLS are emerging targets for manipulation of the immune responses underpinning chronic inflammation.

TLS exhibit almost all the characteristics of the classical secondary lymphoid organs (SLO), but notably lacking a surrounding capsule (2). These structures are associated with the development of a local adaptive immune response. There is no consensus on the minimum size an aggregate must have to be considered a TLS. However, three stages of TLS maturation have been identified. Early-TLS (eTLS) are poorly structured B and T cell aggregates. Primary follicle-like TLS (PFL-TLS) are more strongly organized with clear B cell zone, mostly composed of naïve B cells, supported by CD21^+^ follicular dendritic cells (FDC) without evidence of a germinal center reaction (3). Finally, fully mature secondary follicle-like TLS (SFL-TLS) with a functional germinal center (GC) containing CD21^+^CD23^+^ FDC, proliferating B cells (4, 5), follicular helper T cells and plasma cells, which evidence an active humoral immune response (3). TLS are accompanied by high endothelial venules (HEV) and specialized lymphoid fibroblasts (2, 6).

TLS can be detected using several methods (7), including analysis of hematoxylin and eosin stained tissue sections, immunohistochemistry, or through the expression of various gene signatures. Several such gene signatures have been reported over the years, including a 12-chemokine signature derived from mRNA microarrays (8), an 8-gene signature accounting for the presence of follicular helper T cells (9), a 19-gene signature representing the presence of T helper 1 cells (Th1) and B cells, and expression of the B-cell chemo-attractant CXCL13 (10, 11). More recently, analysis of spatially-resolved transcriptomics data in clear-cell renal cell carcinoma allowed the discovery of a 29-gene signature called “TLS imprint” that is specifically expressed within TLS (12). This signature notably involved genes related to T cells, B cells, immunoglobulins, fibroblasts and complement genes (12).

The clinical impact of TLS highly depends on the type of disease considered. In viral infection, the presence of TLS has a protective role for the host (13–15). In cancer, mature TLS are associated with longer survival of patients in the vast majority of malignancies (16, 17). The presence of TLS has been shown to be associated with an improved response to immunotherapy in soft-tissue sarcoma (11, 18), melanoma (19, 20), clear-cell renal cell carcinoma (12, 20), breast cancer (21) and lung cancer (22, 23). In a pan-cancer setting, it was reported that mature TLS could be used as a predictive biomarker for immunotherapy response (24). In autoimmune diseases, however, TLS are commonly associated with disease severity (25). This is notably the case in Sjögren syndrome (26–29), where TLS have also been associated with a higher risk of B cell lymphoma development. In some other pathologies, such as lupus nephritis and rheumatoid arthritis, the evidence to date also suggests the involvement of TLS in pathogenesis (30).

DCs are professional antigen-presenting cells (APCs) that play a critical and decisive role in determining the outcome of the immune response to antigens, since they determine T cell activation and polarization (30). Broadly, DCs can be classified into several subsets, being the most established ones: conventional DCs 1 (cDCs1) and 2 (cDCs2) and plasmacytoid DCs (pDCs) (30, 31). Conventional DC1 have a high intrinsic capacity to cross‐present antigens via MHC class I to activate CD8^+^ T cells and to promote T helper type 1 (Th1) and natural killer responses. Conventional DC2 potently activate Th2, Th17 and CD4^+^ T cells through MHC class II (30, 32). pDCs produce large amounts of type I interferon (IFN-I), inducing antiviral immune responses (2, 31, 33–35). More recently, additional DC subsets have been reported, including monocyte-related DC3 and cDC2-related DC5 cells (36) but their functions and developmental origins are less explored. In addition, non-hematopoietic FDCs are accessory immune cells in TLS that contribute to the regulation of humoral immunity. They are generally located in the B-cell follicles of the secondary lymphoid tissues where they present antigens to B-cells. These cells are also essential in the induction of the germinal center. FDCs aid in the rescue of B cells from apoptosis and induce the differentiation of B cells into long-term memory B cell clones or plasma cells (37–39).

Here, we conducted a comprehensive review of the relevant literature to analyze the distribution and function of different DC subsets in TLS in cancer, autoimmunity and chronic inflammation. Understanding the roles of these subsets within TLS contributes to elucidate the mechanisms behind disease progression and for identifying potential therapeutic approaches across diverse pathological conditions.

## 2 DCs subtypes in different tumor types

Even if the number of DCs infiltrating the tumor microenvironment (TME) is very low, they play an essential role in mounting anti-tumor immunity. The subsets have specialized but also overlapping roles in the TME, including tumor antigen recognition, immune surveillance, cross-presentation, recruitment and activation of other immune cell types, and induction of tumor-specific CD4^+^ and CD8^+^ T cells. The DC phenotype and function are not static and may depend, for example, on the tumor cells and other immune cells in the TME (cross-talk) and on therapy like immune modulating agents (40). Briefly, pDCs, which in humans express the markers BDCA-2 (CD303) and CD123, can produce large amounts of type I interferons (IFNs) which are important for the generation of immune responses against tumor cells and viruses (35). Human cDC1 (which express the markers CD141^+^XCR1^+^CLEC9A^+^) can present antigens through MHC class II to activate Th1 cells during the early stage of tumor progression or inflammatory conditions (41). These primed/licensed cDC1s will then play an important role in the cross-presentation of tumor antigens to CD8^+^ cytotoxic T cells (CTLs) (42). cDC1 is also involved in the recruitment of CD4^+^ T cells (41) and CD8^+^ T cells to the TME via CXCL9 and CXCL10 (43). cDC2s (identified as CD1c^+^CLEC10A^+^) present extracellular antigens to subsets of CD4^+^ T cells and can secrete IL-12 (44) which is important for the activation of effector cells within the TME. Recently, single-cell RNA sequencing (RNA-seq), multi-color flow cytometry and mass cytometry techniques for high dimensional biomarker analysis of various solid tumor types have revealed distinct DC subsets present in the TME. Apart from pDCs, cDC1 and cDC2s, DC3s have been detected in e.g. breast tumors (45), and head and neck squamous cell carcinoma (HNSC) (46, 47). Human DC3 shares surface markers with cDC2 (CD1c) and monocytes/macrophages (CD14, CD163), and differentiate via a pathway activated by GM-CSF, independent from development of cDCs and monocytes (45). Recent fate mapping and scRNAseq experiments, using murine bone marrow, spleen and blood samples, demonstrate that DC3s arise from a Lyz2^+^ Ly6C^+^CD11c^−^ DC progenitor, called pro-DC3 (48). *In vitro*, activated DC3 can produce high amounts of cytokines (IL-12p70, IL-23, TNF-α) and chemokines (CXCL9, CXCL10, CXCL11, and CCL5), and activate naive T cells as efficiently as cDC2s (45) (49), indicating a role for DC3 in T-cell activation and attraction within the TME. Moreover, DC3s infiltration in human breast tumor have been associated with a subset of tissue resident memory CD8^+^CD103^+^CD69^+^ T cells (45). A recent study showed that non-small cell lung cancer patients exhibit elevated frequencies of peripheral blood CD14^+^ cDC2s, phenotypically resembling DC3s, and that these cells display increased expression of e.g. PD-L1, IDO and IL10, and an impaired potential to activate T cells (49). Other DC subtypes include the DC5, also known as AS-DC, and pre-cDCs which have partially overlapping expression profiles (CD123, AXL, SIGLEC6, CD11c, CD1c) (50). DC5s are efficient at T cell stimulation, have a lower ability to produce IFNα than pDCs (51), and have a morphology similar to cDCs (50). These cell types have been detected by scRNAseq in tumor types such as glioblastoma (pre-cDCs) (52) and tonsillar cancer (DC5) (47). Further, a population, sharing features of cDC2 and expressing markers of monocyte-derived DCs (MoDC) or Langerhans-like cells (CD1a, CD207), has been identified by scRNAseq in several solid tumor types, including lung, colorectal, ovarian, breast (53) and head and neck cancers (47). The functional specialization of DC3, AS-DC/AXL^+^ DCs and MoDCs within the human TME remains to be determined, and it is unclear where these cell types are located and how the diversity is affected by treatment.

Other studies have identified the presence within the TME of mature regulatory DCs (mregDCs), also known as activated DCs, LAMP3^+^ DCs or migratory DCs, characterized by a mature phenotype and markers such as CCR7, DC-LAMP, CCL19 and CCL22, and lacking cell-type specific markers of pDCs, cDC1s, cDC2s or DC3s (54). This population has been identified in a wide range of cancer types, including hepatocellular carcinoma (55), colon cancer (56), tonsillar cancer (47) and lung cancer (57). Tumor infiltrating mregDCs are also characterized by the expression of immunoregulatory markers, such as PD-L1/2, TIM3 and IDO, which limit T‐cell activation (58).

DCs are strongly influenced by both extrinsic and intrinsic factors in the TME, recently reviewed in (59), which affect their phenotype and functional properties. Extrinsic factors include inflammatory or immunosuppressive cytokines (e.g. TNFα, IL-10, TGFβ), viral antigens (e.g. HPV), hypoxia-induced factors (e.g. HIF1α) or damage-associated molecular patterns (DAMPs) released by injured or stressed cells (e.g. ATP, adenosine, DNA/RNA, S100 proteins). Tumor-derived retinoic acid can also direct monocyte-differentiation towards immunosuppressive macrophages rather than immunostimulatory DCs by downregulation of Irf4 (60). Furthermore, tumor cells can exert DCs-escaping strategies, such as secretion of soluble factors (VEGF, PGE2, TGFβ), products of metabolic stress (lipid peroxide), β-catenin production and immune checkpoints expression (PD-L1). In addition, DC-priming fails in tumors with low neoantigens expression resulting in ineffective T cell activation (61). Examples of cell-intrinsic factors include the metabolic checkpoint STING, which restricts aerobic glycolysis to promote antitumor immunity (62), or the Wnt1-β catenin pathway that leads to reduced intratumoral DCs-secreted chemokines in human lung adenocarcinoma samples (63). Further, the presence of DCs in the TME is tightly regulated by chemokine activity. For example, mregDCs express CCR7 within the TME, suggesting migratory potential from tumor sites to lymph nodes towards CCL19 and CCL21-expressing lymphatic endothelial cells or nearby DCs. MregDCs can also express CCL19, CCL21 and CCL22, indicating that they also are involved in recruitment of other lymphocytes expressing CCR7, CCR4 and CCR3 (58). Interestingly, immunohistochemical results suggest that mregDCs expressing LAMP3 and/or PD-L1, accumulate in the TLSs in close contiguity with T cells (6, 64, 65). Within the TME, XCR1^+^, CCR5^+^ cDC1 can be recruited by NK cells expressing XCL1 and CCL5 (66). More studies regarding the migration, and co-localization with other TME cell types, of DCs are warranted, and new technologies based on spatial protein-based or transcriptomic platforms, in combination with artificial intelligence (AI) models, are certainly valuable as they may capture the heterogeneity and plasticity of the DC compartment. One example of models that can be useful for analyzing spatial data is the Spatial Cellular Network (SpaCeNet), which is designed to asses intra- and inter-cellular networks in the TME (67).

## 3 Association of DC subtypes with cancer prognosis

Several studies have shown the association of DC subtypes/states with prognosis in various cancers, based on their frequencies in tissue or transcriptional profiles. Gene signatures of cDC1 have been associated with a favorable prognosis in multiple human cancer types (47, 66, 68), including breast cancer, melanoma, head and neck squamous cell carcinoma, and lung adenocarcinoma. A gene signature of cDC1, including CLEC9A, XCR1, BATF3, and CLNK, has also been shown to be positively correlated with the signature of NK cells in these cancer types (66). In addition to cDC1, pDC and cDC2 gene signatures have been shown to be predictive of disease-free survival in human primary luminal breast cancer (68). It has been shown that cDC1 abundance in human melanoma correlate with T-cell infiltration and further that the ratio of cDC1-selective transcripts over macrophage-restricted transcripts can be used as a prognostic marker for survival (66). Spranger et al. shed light on the mechanism responsible for T-cell infiltration by discovering that CD103^+^cDC1 secreted chemokines (CXCL9, CXCL10) were necessary for the recruitment of T lymphocytes in the TME. Here, the non-T cells inflamed human tumor model characterized by lack of cDC1s cells displayed deficient T cell trafficking within the tumor tissue (43). Barry et al. demonstrated the ability of NK cells to recruit CD141^+^ cDC1s in metastatic melanoma TME through FLT3L cytokine secretion. With this work, the group implied that sustained NK-cDC1 cells axis might favor anti-PD-1 treatment responsiveness and patients survival through the recruitment of CD141^+^ cDC1s cells in the tumor sites (69). The prognostic value of activated or LAMP3^+^ DCs, features of mregDCs, have also been established for many cancer types. For instance, high density of LAMP3^+^ DCs has been associated with longer survival in melanoma (70) and colorectal carcinoma (71), and a 3-gene transcriptional signature (LAMP3, CCL19, CCL22) correlates with increased survival in head and neck cancer (47). pDC infiltration in the TME has been associated with both favorable and worse prognosis in several cancer types. For instance, a higher density of pDCs has been associated with poor outcomes in hepatocellular carcinoma (72). In contrast, a higher density of tumor-infiltrating pDCs has been associated with prolonged survival in colon cancer (34). In the same study, pDCs were preferentially co-localized with CD8^+^ T cells in the stroma and CD4^+^ T-cells in the T-cell zone of colon cancer-associated TLS (34). Recently, increased frequencies of CD14+ cDC2s, resembling DC3 phenotypically, was shown to correlate with reduced survival for melanoma patients receiving CD1c+ DC vaccination (49).

Taken together, these studies demonstrate that the presence of different DC subsets in the TME is correlated with cancer prognosis, with cDC1 and LAMP3^+^ DC mostly associated with a favorable prognosis while the role of other subsets such as pDC remains controversial. More research is necessary to gain insight into the potential of using the presence of DC subsets as biomarkers for predicting disease outcomes and therapeutic responses.

Additional studies investigating the role of specific DCs subsets in low immunogenic tumors are needed. These tumor types are characterized, for example, by defective antigen presentation (73) which results in impaired anti-tumor immune response. Dunne et al., studied a cohort of 152 colorectal cancer patients demonstrating positive correlation between high HLA-DR levels and overall survival in colorectal cancer epithelium, but not in the stroma (74). Furthermore, the group reported a correlation between loss of HLA-DR expression and tumor progression (74), suggesting DCs perpetual impairment within TME during tumor development. Interestingly, Vacchelli et al. reported in human and murine breast and colon cancer models, the presence of defective DCs having a loss of function in the formylpeptide receptor 1 (FRP1) allele. This mutation resulted in anthracyclines-based chemotherapy failure due to impaired recognition of dying tumor cells by DCs (75). Although these studies shed a light on the mechanisms behind antigen presenting cells and DCs dysfunction in low immunogenic tumors, many details regarding specific DCs states in the TME, remain yet to be clarified.

To counteract dysfunctional DC in tumors, intratumoral DC delivery has been investigated to elicit immune cells anti-tumor activity. A phase I trial, in patients with advanced NSCLC utilized *in-situ* injection of autologous DCs transduced with an adenoviral vector expressing CCL21-gene (AdCCL21-DC), aiming at sustaining lymphoid tissue organization and facilitating DCs and T cell interactions. This strategy showed increased CD8^+^ T infiltration after AdCCL21-DC’s local injection and parallel induction of PD-L1 mRNA expression (76). Interestingly, an earlier study has demonstrated that local injections of DCs bearing apoptotic/necrotic B16 in melanoma cells resulted in lymphocyte homing through high endothelial venules (HEV) development at the DCs vaccination site, suggesting the potential of DCs vaccines to induce *de novo* TLS formation (77). These data thus show that the presence of DCs able to support lymphocyte activation, also at the site of the tumor, is highly beneficial to mount effective anti-tumor immune responses.

## 4 Role of DC subsets in TLS formation in cancer

There are only few studies that particularly focused on the role of DCs in tumor-associated TLS besides the role of FDCs (**Table 1**). Nevertheless, the presence of DCs within TLS has been associated with a favorable clinical outcome in patients with non-small cell lung cancer (NSCLC) (78, 79). Lately, agonist strategies of the cGAS/STING pathway to induce type I interferon have been successfully explored both in pre-clinical models and clinical studies in cancers (86–89) (87, 89). Interestingly, a recent study showed that intratumoral injection of a STING agonist in murine subcutaneous melanomas promotes the assembly of TLS, infiltration of CD11c^+^ DCs and slowed tumor growth (89). Further, STING activated CD11c^+^ DCs upregulated expression of TLS promoting factors, including lymphotoxin-α (LTA), IL-36 and type I IFNs (89).

**Table 1:** DC in TLS in cancer.

Pathology	DC Subtype	DC Markers	Methods used for TLS identification
Non-small cell lung cancer (NSCLC) (78, 79)	Mature DCs	DC-LAMP^+^	IHC
Lung Cancer (80)	Mature DCs	LAMP3^+^	IHC
Non-small cell lung cancer (NSCLC) (81)	mregDC	DC-LAMP^+^	Multiplexed immunohistochemical consecutive staining on a single slide (MICSSS)
Soft tissue sarcoma (18)	cDCs	HLA-DR^+^, CD11c^+^, CD1c^+^	IHC
Renal cell carcinoma, Bladder cancer and Prostate cancer (82)	undefined	CD11c^+^MHC-II^+^	IHC
Hepatocellular carcinoma (83)	–	CD208^+^	IHC
	FDCs	CD21^+^	
Non small lung cancer (84)	–	*CCR7* ^+^ *LAMP3* ^+^	
Breast Cancer (85)	–	DC-LAMP^+^ CD11c^+^	IHC
		CD45^+^, lin^−^, HLA-DR^+^, CD11c^+^, CD1A^+^	Flow cytometry with cell sorting

DCs, Dendritic cells; cDCs, Conventional DCs; mregDCs, Mature DCs enriched in immunoregulatory molecules; FDCs, follicular dendritic cells; IHC, Immunohistochemistry.

Summary of studies where DC have been identified associated with TLS in human cancer.

In regard to pDCs, Kießler et al. identified an activated BDCA-2^+^ pDCs state within TLS and stroma tissue of colon cancer patients. Here, the multiplex immunofluorescence staining revealed the presence of IRF7^+^ pDCs mainly in TLS’ T cell zone and in the proximity of stroma’s Granzyme^+^ CD8^+^ T cells (34). These findings suggest that pDCs may stimulate CD8^+^ T cells within tumor’s TLS and represent a predictive marker for favorable clinical outcome (34).

Importantly, mature TLS, characterized by the presence of germinal centers and the presence of CD21^+^CD23^+^ FDC have been shown to be a stronger predictor of response to immunotherapy than overall TLS presence, including TLS lacking FDC (24). In lung cancer, LAMP3^+^ mature DCs have been shown to favor Th1-polarised and cytotoxic T cell responses, thereby favoring longer patient survival (80). It has been hypothesized that this subset of DCs corresponds to mregDC (90). In cervical cancer, recent results indicate that the presence of TLS is associated with a higher presence of DC, and in particular favors cDC2-T cell interactions (91). In lung cancer, mregDC are particularly enriched inside the TLS, where they physically interact with CD4^+^PD-1^+^CXCL13^+^ T cells (81). Importantly, these T cells were found to be clonally expanded, which could indicate that mregDC have presented tumor antigen to them. Mature DC potentially contribute to the beneficial role of TLS with regards to immunotherapy response. Indeed, the in PEMBROSARC clinical trial based on pembrolizumab combined with low-dose cyclophosphamide, in patients with soft-tissue sarcoma selected to all be TLS positive, it was reported that a higher density of CD11c^+^HLA-DR^+^ DCs was associated with an increased progression-free and overall survival (18). This study underlines the TLS’s double edge sword effect, which seems to depend on the frequencies of resident immune cells that populate these structures.

In various malignancies, studies report the presence of less organized immune cell aggregates, that do not fit the definition of TLS. DC have been reported in some of these, but it remains unclear whether these would evolve into TLS or not, and they have a varying prognostic impact (92). In renal cell carcinoma, bladder cancer and prostate cancer, these aggregates, labeled Antigen-Presenting Cell niches, exhibit colocalization of MHCII^+^ antigen-presenting cells of unspecified nature and TCF1^+^CD8^+^ T cells (82). They are present in the tumors of patients that have lower progression-free survival. In hepatocellular carcinoma, TLS with a higher density of immune cells, including DC, have been correlated with a better prognosis (83). Most recently, immune triads grouping mregDC, CD4^+^CXCL13^+^ helper T cells, TCF1^+^PD-1^+^CD8^+^ T cells and B cells were identified (93). These immune triads are more common in tumors of patients responding to PD-1 inhibitors. In non-small-cell lung cancer, so-called stem immunity hubs containing mregDC adjacent to CD4^+^ T cells, some of which are Treg, with CXCL10^+^ macrophages and TCF7^+^PD-1^+^CD8^+^ T cells were recently shown to associate with better response to PD-1 blockade (84, 94). In breast cancer, aggregates of B, T and DC have been reported, with LAMP3^+^ DC clusters contributing to the density of HEVs and clinical outcomes (85).

In summary, a few studies have analyzed DCs within tumor-associated TLS, with the presence of DCs being linked to favorable clinical outcomes. Mature TLS seem to be good predictors of positive immunotherapy responses. Interestingly, STING agonists are potential modulators of TLS, and may contribute improve cancer treatment outcomes. Specific DC subsets, such as LAMP3+ mature DCs and mregDC, have been associated with enhanced Th1 and cytotoxic T cell responses, contributing to improved survival rates. Overall, the interaction between DCs and other immune cells within TLS or immune aggregates warrants further investigation, since it presents a promising avenue for future therapies.

## 5 DCs subtypes in autoimmune and inflammatory conditions

Autoimmunity occurs when there is a breakage of self-tolerance mechanisms, leading to the immune system attacking the self. In contrast, inflammatory conditions result from chronic, uncontrolled inflammation, which may occur independently of self-reactive T cells or antibodies. DCs mediate immunogenicity, as they promote the activation of adaptive immunity but are also capable of inducing tolerance (95, 96). These cells work as the link between innate immunity that leads to initiation of adaptive immunity through presentation of antigens to T cells. Simultaneously, these cells can present self-antigens or innocuous antigens to T cells without costimulation and/or activating cytokines, leading to the development of tolerance mechanisms. Thus, it has been proposed that tolerogenic DC or DC-targeting nanomedicines may be used as therapeutics against autoimmune diseases such as type 1 diabetes (97, 98). On the other hand, dysregulations in a balanced DC response can contribute to the onset and development of both autoimmune and chronic inflammatory pathologies (99). DC can capture and process antigens from self-tissues and potentially present self-antigens to autoreactive CD8^+^ T cells, contributing to autoimmune responses with destruction of the host cells, or to CD4^+^ T cells, priming Th lineages, while also promoting production of pro-inflammatory cytokines that can exacerbate autoimmune responses (96, 100). Broadly, cDC1 can activate CD8^+^ T cells, while cDC2 can trigger CD4^+^ T cells and it is their concerted action that is likely to drive (auto)immunity (96). Consequently, different subsets of DC have been reported in autoimmune and inflammatory conditions. For example, in Type 1 Diabetes, cDC1 can activate autoreactive T cells, contributing to the destruction of pancreatic β-cells (101, 102). In human patients with IgA nephropathy and lupus nephritis, there is an increased number of both CD8^+^ T cells and cDC1, which correlated with occurrence of interstitial fibrosis, suggesting that interactions between cDC1 and CD8^+^ T cells contribute to pathogenesis (103). On the other hand, cDC2s have been found in the synovial fluid and tissue of patients with rheumatoid arthritis (104, 105). pDC have also been reported to contribute to autoimmune diseases, in this case, Systemic lupus erythematosus (SLE), Systemic Sclerosis or type I diabetes (106, 107). When dysregulated, these cells drive autoimmunity by producing IFN-I, upon detection of immune complexes formed with autoreactive antibodies, thus promoting the pathology in a positive feedback loop. Moreover, inflammatory DC3, identified as CD5^-^CD163^+^ cells, are expanded in blood samples from patients with SLE and correlate with disease activity, with a correlation between the proportion of circulating CD163^+^ DC3s and the SLE disease activity index (36). Furthermore, these cells were found to express increased amounts of interferon-stimulated genes and inflammatory genes, showing strong activation of the death receptor signaling and DC maturation pathways when comparing SLE patients with healthy donors. Moreover, it was found that CD163+ DC3 secrete increased amounts of pro-inflammatory cytokines when incubated with serum from SLE patients. All these alterations occurred specifically in CD163+ DC3, and not on other DC subsets, suggesting that these cells are significant contributors to disease pathogenesis (36). Finally, mregDCs and CD14^+^ DC3 cells have also been reported in autoimmunity, in patients with atopic dermatitis or psoriasis (108).

## 6 Role of DC subsets in TLS formation in autoimmune and inflammatory conditions

TLS have been reported in autoimmune conditions, and harbor autoreactive B and T cells (**Table 2**). A key feature of autoimmunity is the activation of B cells, as they produce autoantibodies, which target and react against self-structures (113). Simultaneously, autoreactive T cells will contribute to exacerbated production of inflammatory cytokines and tissue damage (114).

**Table 2:** DC in TLS in autoimmune and inflammatory conditions.

Pathology	DC Subtype	DC Markers	Methods used for TLS identification
Inflammatory bowel diseases (IBD) (109)	Myeloid DCs	CD11c^+^	IHC
	Immature DCs	CD1a^+^	
	Mature DCs	CD83^+^	
	pDCs	BDCA‐2^+^/CD123^+^	
Inflammatory bowel diseases (IBD) (3)	FDCs	CD21^+^CD23^+^	IHC
	Mature DCs	DC-LAMP^+^	
Chronic Obstructive Pulmonary Disease (COPD) (110)	cDC1s	CD141^+^	Single-Cell RNA-Sequencing and Flow Cytometry
	cDC2s	CD1c^+^	
IgA nephropathy (111)	DCs and FDCs	DC-SIGN^+^	IHC
Rheumatoid Arthritis (112)	FDCs	CD21^+^	IHC

DCs, Dendritic cells; pDCs, Plasmacytoid dendritic Cells; FDCs, follicular dendritic cells; IHC, Immunohistochemistry.

Summary of studies where DC have been identified associated with TLS in human autoimmune and inflammatory conditions.

It has been suggested that there is reduced selection of autoreactive B cells in TLS in comparison with secondary lymphoid structures (114, 115). At the same time, TLS are more exposed to the surrounding microenvironment, and thus may be more effective in initiating an immune response than encapsulated secondary organs. Consequently, TLS may be important contributors for autoimmunity, with the production of disease-specific autoantibodies in TLS associated with target organs (25). TLS can also have a role in autoimmune diseases with auto-reactive T cells. In multiple sclerosis, autoreactive T cells target myelin antigens, leading to neurodegeneration. TLS have been reported in the meninges and associated with sub-pial cortical damage and disease progression (116). Apart from T cells, these meningeal TLS can also contain proliferating B cells and plasma cells, but the role of these TLS in antibody class switching remains unclear (117). Using the experimental autoimmune encephalitis (EAE) mouse model, T helper 17 (Th17) cells have been associated with the formation of TLS in the brain meninges and to contribute to local demyelination, astrogliosis and complement deposition. In addition, the coordination between Th17 cells and the lymphotoxin pathway leads to local cytokine production and Th17 cell responses (118).

TLS are found also in regions other than local organs, such as the thymus. For example, in myasthenia gravis, where patients exhibit the presence of anti-nicotinic acetylcholine receptor (AChR) antibodies, causing a severe impact in neuro-muscular communication, TLS have been detected within the thymus (119). It has been suggested that these ectopic structures are correlated with anti-AChR antibody production triggered by intrathymic T follicular helper (Tfh)/B cell interaction (119). And interestingly, a recent study found a correlation between the presence of Germinal Centers in thymomas that were surgically removed from patients and the postthemectomy development of MG (120). These studies suggest that Tfh – B cell interactions may contribute for auto-antibody production and MG onset.

In inflammatory conditions, there is a perpetuation of chronic, detrimental inflammation in response to antigens that would normally pose little or no threat. Since TLS can locally facilitate interactions and activation of immune cells, they can amplify the immune response at the site of inflammation, resulting in a sustained immune response (6, 114, 121–123). Indeed, TLS have been described in several pathologies where autoimmune and inflammatory reactions occur, and their presence and maturation stated has been associated with the prognosis, albeit with some contradictory results (114).

Few studies have analyzed the role of DC in activating immune responses within TLS. The existence of FDC has been documented, which will be important for B cell activation, and production of auto-antibodies. However, only a few studies analyzed other DC subsets and most simply describe TLS and correlate their presence and/or maturation state with disease prognosis. Thus, there is still a major lack of knowledge on the role played by DC within TLS in the context of autoimmunity and inflammatory conditions, and also on tools with the potential to manipulate these structures in clinical settings.

### 6.1 TLS in chronic inflammation

Inflammatory bowel diseases (IBD) are a group of diseases represented by Crohn’s disease (CD) and ulcerative colitis (UC), characterized by uncontrolled and chronic intestinal inflammation with multifactorial pathogenesis (124–126). In UC, the inflammation is restricted to the colon, primarily in the mucosa and to a lesser extent in the submucosa. CD is characterized by segmental involvement of the entire gastrointestinal tract, with transmural inflammation, although the most commonly affected site is the terminal ileum. An important hallmark of CD is the formation of epithelioid granulomas (124, 127). According to current hypotheses, chronic inflammation results from a dysregulated immune response to commensal bacteria that penetrate the bowel wall due to impaired intestinal barrier function (124). The ensuing immune activation is associated with release of inflammatory mediators that leads to epithelial damage and exacerbate and propagate the gut inflammation (126, 128). The mechanisms that promote the B and T cell-directed anti-microbial immune responses in IBD are poorly understood. However chronic intestinal inflammation is associated with a large expansion of the intestinal lymphoid tissue network (129–131), including structures of the gut-associated lymphoid tissues that develops during development, and TLS induced by inflammation (124).

In support of this, a study showed that TLS were present in surgically resected intestine in over 90% of patients with CD (132). These TLS are present within all layers of the intestine, including the deep layers, and may have different levels of organization (124). TLS-like structures have also been detected in the mesentery of the intestine and the surrounding enlarged lipomatous tissue, described as “creeping” fat, in patients with CD (133–135). It is thought that the formation of these structures in CD is the result of the action of, at the very least, pathogen-derived stimuli and TNF-α. TLS localization corresponds to areas with high local production of chemokines such as CCL19, CCL20, CCL21, CXCL13, and CXCL16, probably produced by adipocytes (135). TLS in the mesentery are suggested to both mediate local immunity (134), and, participate in remodeling of collecting lymph vessels, which may obstruct the lymphatic system (133). A dysfunctional lymphatic system may contribute to impaired trafficking of DC to mesenteric lymph nodes, which was reported to compromise normal mucosal immune functions, such as tolerance, contributing to dysregulated inflammation in the intestine (136, 137). DC are found in increased numbers in tissues affected by CD, compared with unaffected tissues (intermittent non-inflamed colonic tissue from CD patients, diverticulitis, and non-inflammatory gut disorders), based on the expression of fascin (109). Most of these cells (70%-80%) are mature myeloid DC, according to their expression of CD83, and are located in the proximity of proliferating T-cell clusters indicating their antigen-presenting function in tissue affected by CD (109)., Furthermore, increased expression of ligands CCL19 and CCL21 chemokines, as well as their receptor CCR7 in tissues affected by CD was shown (109). This may indicate that DCs are retained in the CD lesions, and thus, participate in development of TLS (109). These findings imply that in inflamed tissues, a chemokine environment similarly to the one present in secondary lymphoid tissues, is created, promoting DC/T cells interaction and, thus, initiation and expansion of (autoreactive) T cells (109).

More recently, single-cell analysis of intestinal samples from CD-patients revealed an expansion of activated LAMP3+ DCs in subjects with treatment-resistant disease, which was associated with a cell module comprising IgG-producing plasma cells, activated T cells and myeloid cells, as well as inflammatory fibroblasts (138). Such DCs were found in mucosal aggregates with T and B cells, resembling TLS. Another study reported that, in patients with IBD, mature, DC-LAMP^+^, DC can be found in T cell zones in three different stages of TLS maturation, namely lymphoid-cell aggregates, non-GC TLS and GC-like TLS (3). Regarding the role of TLS in IBD, although it is unclear whether they may have protective or deleterious impact, association of these structures with severe form of CD suggests the later (135, 139). Recently, results obtained in a T cell transfer animal model of colitis suggest reciprocal regulation of Tfh and DC in colonic lymphoid follicles within TLS (140). It was shown that Tfh cells impact accumulation of mature DC in lymphoid follicles, and on the other hand, mature DC promote Tfh differentiation into pathogenic Th1 cells, contributing to progression of colitis (140).

Chronic Obstructive Pulmonary Disease (COPD) is a chronic lung disease with a strong inflammatory response characterized by progressive airflow limitation and tissue destruction. In COPD the development of TLS is related to the severity of the disease and tissue destruction (141–143). A study that analyzed human lung samples from COPD patients proposed a role for cDC2 in TLS formation (110). Results showed that cDC2s from COPD patients express unique migratory signature with increased expression of CXCR4, CXCR5 and Epstein-Barr virus–induced gene 2 (EBI2) molecules. These molecules enable them to migrate to TLS, attracted by CXCL12, CXCL13 and oxysterol (cholesterol metabolite) which are increased in lungs of COPD patients. Furthermore, cDC2 cells in COPD are potent in skewing naïve CD4+ T cells to IL-21- and CXCR3- secreting Tfh cells through the OX40L-OX40 interaction, thus being involved in the formation and maintenance of the established TLS (110).

In summary, available literature data implies that TLS have a role in pathogenesis of chronic inflammatory diseases, but it is unclear whether the role is deleterious or beneficial. In favor of deleterious effects are findings that: a) in IBD and COPD the presence of these structures correlates with more severe forms of diseases; b) in these diseases, the DC found within TLS have a mature phenotype and are in close proximity to activated T cells, myeloid cells, inflammatory fibroblasts, and IgG-producing plasma cells, and have the ability to polarize Tfh cells. There findings support the assumption that TLS (and probably DC within these structures) have a role in perpetuation of inflammation. On the other hand, TLS formation may be influenced by microbial dysbiosis, and may exert beneficial effects, in COPD, by establishing control over pulmonary microbiome (121), or rebuilding tolerance to gut microbiome in IBD (124). In the end, that the clinical significance of TLS will likely depends on cellular composition and the stage of disease (114).

### 6.2 TLS in autoimmune diseases

SLE is an autoimmune disease with multi-organ involvement characterized by the presence of anti-nuclear autoantibodies, presenting symptoms associated with accumulation of immune complexes. One of the most serious complications associated with SLE is Lupus nephritis (LN). TLS have been found in the kidneys of patients with SLE and LN and in murine models of these diseases. In a Lupus-prone mouse model, TLS that form in the kidney have a gene expression profile similar to lymph nodes, having FDC in contact with B cells and MIDC-8^+^ DC within T cell areas (144). TLS have also been detected in the pancreas of lupus-prone mice by single photon emission computed tomography (SPECT) upon intraperitoneal injection of 99mTC labelled Albumin Nanocoll and confirmed by immunohistochemistry (145) In humans, it has been found that LN patients have TLS in different maturation states, with its presence being correlated with levels of serum CXCL13, which might suggest that CXCL13 produced by FDC might be involved in TLS development (146). Together, these data point to the role of DC as promoter of TLS development, leading to local activation of adaptive immunity.

IgA nephropathy, or Berger’s disease, is an autoimmune disease where IgA antibodies accumulate in the kidneys, leading to its dysfunction. TLS have also been identified in kidneys of patients with this condition (111). The presence of TLS, identified as organized infiltrates with CD4^+^, CD8^+^ and CD20^+^ cells together with DC-SIGN^+^, scattered in the periphery of the TLS, was correlated with elevated levels of serum creatinine and renal lesions. Moreover, a higher percentage of patients with TLS was detected in a group of patients identified as severe group, with increased serum creatinine >25% above baseline values through the follow-up of 30-months, high levels of heavy proteinuria, serum creatinine, and serum uric acid, a high percentage of mesangial hypercellularity and arterial hyalinosis, severe global glomerulosclerosis, tubular atrophy/interstitial fibrosis, and arterial wall thickening and severe interstitial infiltration of immune cells (111).

Rheumatoid arthritis (RA) is an autoimmune disease characterized by the presence of antibodies in the joints, which leads to severe chronic inflammation, associated with Th1 and Th17 T cell responses. TLS have been found in the inflamed synovium of approximately 50% of RA patients (112), and their presence is correlated with more severe joint pain and systemic inflammation. Human studies suggested that CXCL13 production by FDC is likely to recruit B cells and thus promote the formation of GC in the synovium of RA patients (147). In a study where RA synovium was transplanted into SCID mice, it was found that TLS containing FDC and GC can induce B cell proliferation, class switch recombination and the local production of autoantibodies, thus directly contributing to the damage observed in this pathology (112).

Sjögren’s syndrome is another autoimmune disease with excessive production of autoantibodies, which primarily targets the salivary and lacrimal glands. Interestingly, TLS have been found in biopsies of these affected organs. Some of these TLS exhibit GC and FDC networks, along with the expression of activation-induced cytidine deaminase (AID), suggesting that class switch recombination and somatic hypermutation occur within these specialized locations (148).

Broadly, TLS play a significant role in autoimmune diseases by contributing to immune activation, auto-antibody production, and perpetuation of inflammation. In SLE, LN and IgA nephropathy TLS can be found in the kidneys, and are associated with local immune responses clinical outcomes. In RA, TLS in the synovium are connected to more severe inflammation and local autoantibody production. In Sjögren’s syndrome, TLS in the salivary and lacrimal glands seem to facilitate class switch recombination, further driving the autoimmune response.

## 7 Conclusion and future perspectives

**Figure 1:**
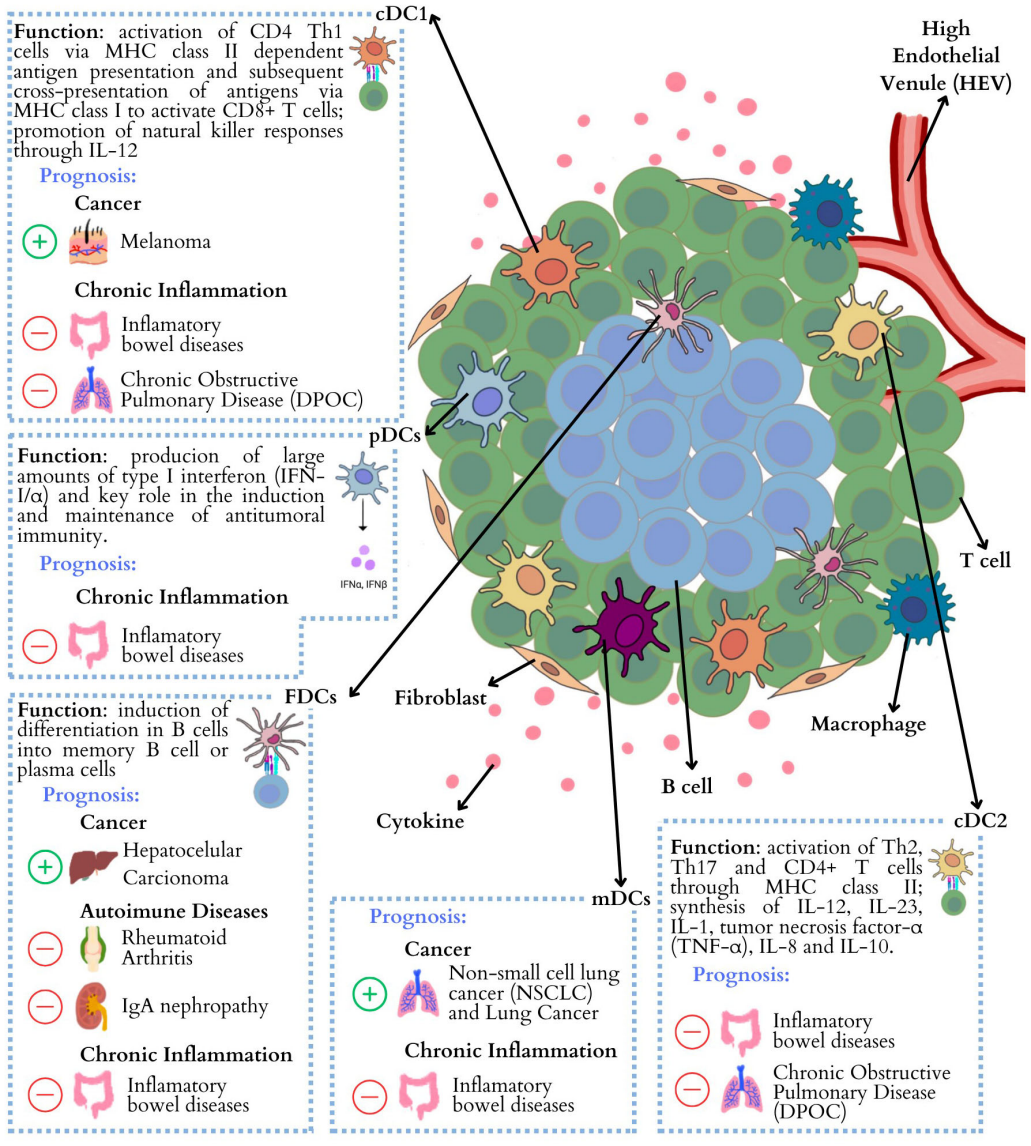
Summary of different Dendritic Cells that have been identified in TLS, and correlated with patient prognosis across cancer, autoimmune and inflammatory conditions (3, 66, 78, 79, 83, 109–112). cDC1, Conventional Dendritic cells type 1; pDCs, Plasmacytoid dendritic Cells; FDCs, follicular dendritic cells; mDCs, Mature Dendritic Cells; cDC2, I Conventional Dendritic cells type 2.

The multifaceted interplay between DCs and TLS presents a complicated landscape in cancer and autoimmune diseases (**Figure 1**). On the one hand, the presence of DCs within TLS appears to be associated with favorable clinical outcomes in various cancers, including non-small cell lung cancer and melanoma. Especially mature TLS, which are characterized by the presence of germinal centers and specific DC subsets, have been identified as a reliable indicator of improved responses to immunotherapy. Additionally, the distribution of distinct DC subtypes within tumor microenvironments, including pDCs, cDC1, and mregDCs, is closely linked to anti-tumor immunity, while the contribution of cDC2 is less clear, emphasizing the diverse roles of DCs in shaping the tumor landscape. In autoimmune diseases, like SLE and RA, the presence of TLS with DC involvement is often associated with increased activity and disease severity. Dysregulated DC responses, particularly cDC1 and cDC2, contribute to autoimmune pathology by presenting self-antigens and inducing inflammation. Furthermore, the role of TLS in autoimmunity goes beyond local tissues, as seen in diseases like multiple sclerosis and myasthenia gravis, where TLS formation in the meninges and thymus correlates with disease progression.

Looking ahead, more research in this field will be required to fully understand the complex mechanisms influencing DC involvement within the TLS (**Table 3**). Deciphering the heterogeneity and functional specialization of DC subsets in different TLS maturation status is crucial. Furthermore, it would be important to evaluate the cross-talk between the DC subsets within the surrounding environment of TLS. Recent technologies, including spatial protein-based or transcriptomic platforms, could provide valuable insights into the dynamic interactions and plasticity of the DC compartment within and around TLS. Moreover, in the clinical setting, manipulating these structures presents a promising path for therapeutic interventions. Targeting specific DC subsets within TLS holds potential for modulating immune responses in both cancer and autoimmune diseases. Nonetheless, the dual function of TLS, acting as both protectors and promoters of disease, underscores the need for careful consideration in therapeutic design. Future research must explore strategies to utilize the beneficial aspects of DC-TLS interactions while minimizing their detrimental effects in autoimmune conditions. In autoimmune disorders that do not respond to traditional immunosuppressors, targeting immune cells, including DCs, within TLS may offer potential therapeutic benefits. Therefore, the continuous effort to analyze the complex functions of DCs within TLS not only enhances our fundamental understanding of immune regulation but also leads to innovative clinical interventions designed for the specific needs of patients with diverse pathological diseases.

**Table 3:** Open questions on the role of DC in TLS in cancer and autoimmune and autoinflammatory conditions.

Open questions
What triggers recruitment/differentiation of different DC subsets to TLS?
How do different DC subsets affect maturation of TLS in different pathological conditions?
How do different DC subsets affect the function of TLS in different pathological conditions?
How can we target specific DC subsets within the TLS?
