# Corrigendum: The role of dendritic cells in tertiary lymphoid structures: implications in cancer and autoimmune diseases

**DOI:** 10.3389/fimmu.2025.1538818

**Published:** 2025-02-07

**Authors:** Mariana Reste, Kristi Ajazi, Ayca Sayi-Yazgan, Radmila Jankovic, Biljana Bufan, Sven Brandau, Espen S. Bækkevold, Florent Petitprez, Malin Lindstedt, Gosse J. Adema, Catarina R. Almeida

**Affiliations:** ^1^ Institute of Biomedicine (iBiMED), Department of Medical Sciences, University of Aveiro, Aveiro, Portugal; ^2^ Department of Immunotechnology, Lund University, Lund, Sweden; ^3^ Department of Molecular Biology and Genetics, Faculty of Science and Letters, Istanbul Technical University, Istanbul, Türkiye; ^4^ Department of Life Sciences, Centre for Inflammation Research and Translational Medicine, College of Health and Life Sciences, Brunel University London, Uxbridge, United Kingdom; ^5^ Faculty of Medicine, Institute of Pathology, University of Belgrade, Belgrade, Serbia; ^6^ Department of Microbiology and Immunology, University of Belgrade - Faculty of Pharmacy, Belgrade, Serbia; ^7^ Experimental and Translational Research, Department of Otorhinolaryngology, University Hospital Essen, Essen, Germany; ^8^ Department of Pathology, Oslo University Hospital-Rikshospitalet, Oslo, Norway; ^9^ Centre for Reproductive Health, Institute for Regeneration and Repair, University of Edinburgh, Edinburgh, United Kingdom; ^10^ Radiotherapy & OncoImmunology Laboratory, Department of Radiation Oncology, Radboud University Medical Center, Nijmegen, Netherlands

**Keywords:** tertiary lymphoid structures (TLS), tertiary lymphoid organs (TLO), dendritic cells (DC), anti-tumor immunity, autoimmunity

In the published article, there was an error in the **Funding** statement. The section originally stated that “COST is supported by the EU Framework Program Horizon 2020”, while it should refer to “Horizon Europe”. The correct **Funding** statement appears below.

“The author(s) declare financial support was received for the research, authorship, and/or publication of this article. This work was developed within the scope of projects with references UIDB/04501/2020 and https://doi.org/10.54499/UIDB/04501/2020, UIDP/04501/2020 and https://doi.org/10.54499/UIDP/04501/2020, 2022.03217.PTDC and DOI 10.54499/2022.03217.PTDC, financially supported by national funds (OE), through FCT - Fundação para a Ciência e Tecnologia, I.P./MCTES. This work was also supported by the World Scleroderma Foundation and Edit Busch Stiftung (MAPFib). This work has been supported by Ministry of Science, Technological Development and Innovation, Republic of Serbia through Grant Agreement with University of Belgrade, Faculty of Medicine No: 451-03-66/2024-03/200110. This work was funded by the Ministry of Science, Technological Development and Innovation, Republic of Serbia through Grant Agreement with University of Belgrade-Faculty of Pharmacy No: 451-03-47/2023-01/200161. This work was supported by the Wellcome Trust (225021/Z/22/Z). This work was supported by the Swedish Cancer Society (22 2221.Pj.01.H) and Mrs. Berta Kamprad’s Cancer Foundation (FBKS-2022-8-368). This work was supported by the Scientific and Technological Research Council of Turkey- TUBITAK (119S447 and 22AG077). This work was also supported by European Cooperation in Science and Technology (COST) Action CA20117 Mye-InfoBank (www.mye-infobank.eu); COST is supported by the EU Framework Program Horizon Europe.”

The authors apologize for this error and state that this does not change the scientific conclusions of the article in any way. The original article has been updated.

